# MBL-Mediated Opsonophagocytosis of *Candida albicans* by Human Neutrophils Is Coupled with Intracellular Dectin-1-Triggered ROS Production

**DOI:** 10.1371/journal.pone.0050589

**Published:** 2012-12-11

**Authors:** Dongsheng Li, Bilin Dong, Zhongsheng Tong, Qinning Wang, Weihuang Liu, Yan Wang, Wei Liu, Jinbo Chen, Li Xu, Liuqing Chen, Yiqun Duan

**Affiliations:** 1 Center for Infectious Skin Diseases, Department of Dermatology, No.1 Hospital of Wuhan, Wuhan, China; 2 Centre for Infectious Diseases and Microbiology Laboratory Services, University of Sydney, Westmead Hospital, Westmead, Australia; 3 Medical Research Center of Wuhan University, Wuhan, China; 4 Institute of Hydrobiology, Chinese Academy of Sciences, Wuhan, China; Academic Medical Center, The Netherlands

## Abstract

Mannan-binding lectin (MBL), a lectin homologous to C1q, greatly facilitates C3/C4-mediated opsonophagocytosis of *Candida albicans* (*C. albicans*) by human neutrophils, and has the capacity to bind to CR1 (CD35) expressed on circulating neutrophils. The intracellular pool of neutrophil Dectin-1 plays a critical role in stimulating the reactive oxygen species (ROS) generation through recognition of *β*-1,3-glucan component of phagocytized zymosan or yeasts. However, little is known about whether MBL can mediate the opsonophagocytosis of *Candida albicans* by neutrophils independent of complement activation, and whether MBL-mediated opsonophagocytosis influence the intracellular expression of Dectin-1 and ROS production. Here we showed that the inhibited phagocytic efficiency of neutrophils as a result of blockage of Dectin-1 was compensated by exogenous MBL alone in a dose-dependent manner. Furthermore, the expressions of Dectin-1 at mRNA and intracellular protein levels were significantly up-regulated in neutrophils stimulated by MBL-pre-incubated *C. albicans*, while the expression of surface Dectin-1 remained almost unchanged. Nevertheless, the stimulated ROS production in neutrophils was partly and irreversibly inhibited by blockage of Dectin-1 in the presence of exogenous MBL. Confocal microscopy examination showed that intracellular Dectin-1 was recruited and co-distributed with ROS on the surface of some phagocytized yeasts. The *β*-1,3-glucanase digestion test further suggested that the specific recognition and binding site of human Dectin-1 is just the *β*-1,3-glucan moiety on the cell wall of *C. albicans*. These data demonstrate that MBL has an ability to mediate the opsonophagocytosis of *Candida albicans* by human neutrophils independent of complement activation, which is coupled with intracellular Dectin-1-triggered ROS production.

## Introduction

Extensive use of broad-spectrum antibiotics, glucocoticoid and immunosuppressant increased incidence of nosocomial fungal infections in immunocompromised patients in the hospital [1]. Candidaemia has ranked as the fourth factor of death caused by blood infection and its gross mortality rate in 30 days nearly accounts for 36.7% [2]. Moreover, *Candida albicans* (*C. albicans*) has been widely considered as the main culprit associated with candidaemia [1]–[3].

The neutrophils, sentinels in human peripheral blood, play a pivotal role in the phagocytosis and fungicidal process upon the occurrence of candidaemia [4]. Furthermore, it is established that the mannan-binding lectin (MBL), a C-type or Ca^2+^-dependent lectin in the serum, has the ability to specifically recognize the mannose moiety of fungal cell wall, and greatly facilitate the C3/C4-mediated opsonophagocytosis of yeasts by neutrophils, together with MBL-associated serine proteases (MASP) [5], [6]. Therefore, the MBL is considered to be important for host resistance to hematogenously disseminated candidiasis [5], [6].

Interestingly, recent studies have further showed that MBL, a lectin homologous to C1q, is known to have opsonic function and can bind to cellular CR1 (CD35) expressed on circulating B lymphocytes, monocytes, and neutrophils, which suggests that neutrophil CR1 may function as a cellular receptor for the collectin MBL [7], [8]. However, little is known about the MBL-mediated opsonophagocytosis of yeasts by neutrophils independent of complement activation till now.

Dectin-1, as a signaling non-TLR pattern-recognition receptor (PRR), shows features of a kind of type II transmembrane receptor that contains a single C-type lectin domain (CTLD) in the extracellular region and an immunoreceptor tyrosine-based activation (ITAM)-like motif within its intracellular tail [3], [4], [9]–[12]. The receptor is expressed widely on innate immune cells including dendritic cells, monocytes/macrophages and neutrophils [3], [4], [9]–[13]. Its specific recognition of *β*-1, 3-glucans, the moiety of fungal cell wall, contributes to the non-opsonic phagocytosis of zymosan or live yeasts by macrophages, and subsequent induction of reactive oxygen species (ROS) partly through Syk-mediated signaling pathway [3], [10], [12], [14], [15].

However, *in vitro* studies pointed out that human neutrophils can effectively phagocytize serum-opsonized zymosan or *C. albicans* independent of Dectin-1 [4].

Noticeably, intracellular pool of neutrophil Dectin-1 was mainly detected in azurophilic granules, in which myeloperoxidase (MPO) was enriched and played a critical role in stimulating the ROS generation [4], [16]. And further study suggested that intracellular expression of Dectin-1 might be involved in the ROS production during the process of fusion between the azurophilic granules and zymosan-containing phagosomes [4].

Therefore, although the phagocytosis of serum-opsonized *C. albicans* by human neutrophils has been demonstrated to be independent of Dectin-1 [4], we can infer that the intracellular Dectin-1-triggered ROS production might be associated with the opsonophagocytosis of *C. albicans* via receptors other than Dectin-1. In this study, we hope to investigate the role of MBL in mediating the opsonophagocytosis of *C. albicans* by neutrophils independent of complement activation, and further to evidence its coupling with intracellular Dectin-1-triggered ROS production.

## Results

### Purity of the isolated neutrophils and FITC-*C. albicans*

**Figure 1:**
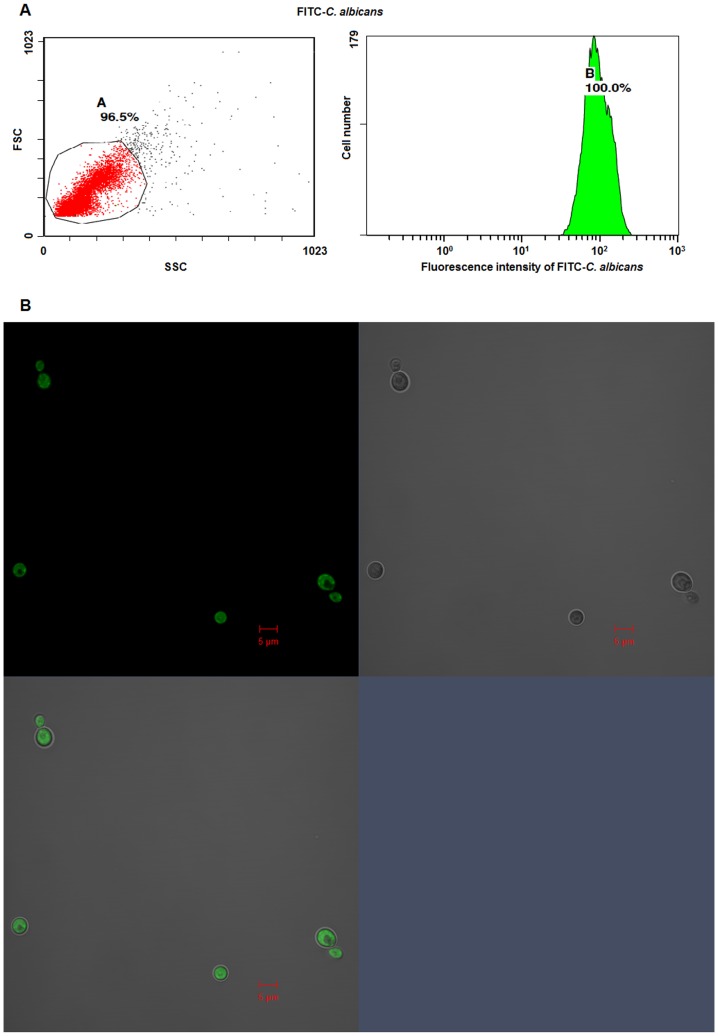
Fluorescence intensity of FITC-*C. albicans* was homogeneously distributed. **A.** FITC-*C. albicans* was selected according to side scatter (SSC) and forward scatter (FSC). Fluorescence intensity of FITC-*C. albicans* was detected by flow cytometry. **B.** FITC-*C. albicans* was examined by Laser Confocal microscopy (10×100). The merged image (left below) showed the distribution of FITC on the *C. albicans*. Scale bar, 5 µm.

Wright stain showed that the purity of the isolated neutrophils was more than 95% pure. Flow cytometry assay confirmed that the intensity distribution of FITC-*C. albicans* was homogeneous and centralized at 10^2^ (Figure 1A and 1B).

### The inhibited phagocytic efficiency of human neutrophils by Dectin-1 blockage was compensated by exogenous MBL

**Figure 2:**
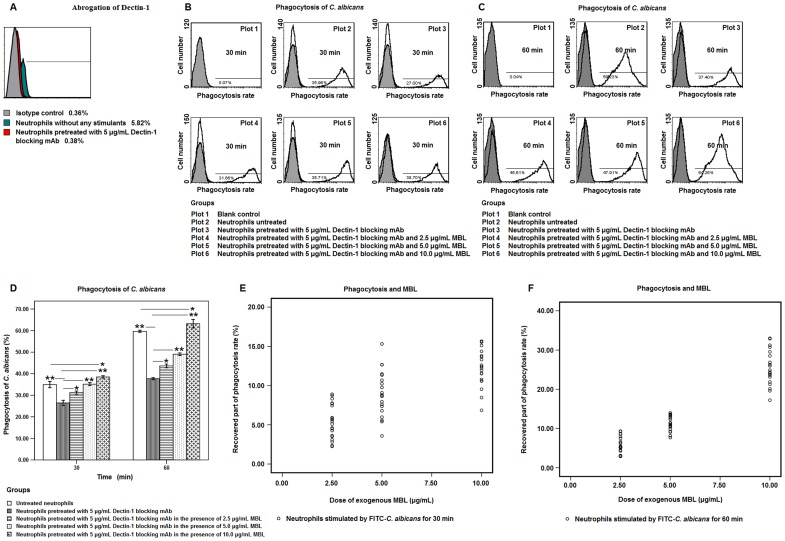
The inhibited phagocytic efficiency of human neutrophils by blockage of Dectin-1 was compensated by exogenous MBL. **A.** Abrogation effect of Dectin-1 on human neutrophils by 5 µg/mL blocking mAb was measured by flow cytometry. PE-mouse IgG2b was used as isotype control. **B** and **C.** Neutrophils containing intracellular FITC-*C. albicans* had distinctive green fluorescence, and were easily differentiated from the ones without intracellular FITC- *C. albicans*. Accordingly, the phagocytic efficiency of neutrophils was measured by flow cytometry assay after stimulation with FITC-*C. albicans* for 30 and 60 min in the presence of 5 µg/mL Dectin-1 blocking mAb and exogenous MBL at a series of concentrations of 2.5, 5 and 10 µg/mL. **D.** Bar graph depicted the phagocytic efficiency of human neutrophils at 30 or 60 min after stimulation by FITC-*C. albicans* in the presence of 5 µg/mL Dectin-1 blocking mAb and exogenous MBL at a series of concentrations of 2.5, 5 and 10 µg/mL. Data were represented as mean ± SE (n = 20). * Significant (<0.05), ** highly significant (<0.01). **E** and **F.** Linear regression analysis between the phagocytic efficiency of human neutrophils at 30 and 60 min after stimulation by FITC-*C. albicans*, and the dosage of exogenous MBL. ** Highly significant (P<0.01).

When the neutrophils were pretreated with 5 µg/mL of blocking antibody, the expression of Dectin-1 was completely abrogated (Figure 2A). As shown in Figure 2B and 2C, the neutrophils which phagocytized FITC-*C. albicans* can be clearly differentiated from the initial ones. Statistical analysis showed that the phagocytic efficiency of neutrophils at 30 min or 60 min after stimulation in the abrogation group was significantly lower than that in untreated group (P<0.01, Figure 2D). However, the phagocytic efficiency of neutrophils at 30 or 60 min after stimulation was completely recovered in the presence of 5 µg/mL Dectin-1 blocking mAb with the adding of 10 µg/mL exogenous MBL, statistically higher than that in untreated group (P<0.05, Figure 2D). Linear regression analysis further indicated that the inhibited phagocytosis rate as a result of Dectin-1 blockage was partly or completely recovered by exogenous MBL in a dose-dependent manner (at 30 min, R^2^ = 0.547, P<0.05, Figure 2E; at 60 min, R^2^ = 0.881, P<0.01, Figure 2F).

### MBL*-*pre-incubated *C. albicans* stimulated mRNA and intracellular expression of Dectin-1 in human neutrophils

**Figure 3:**
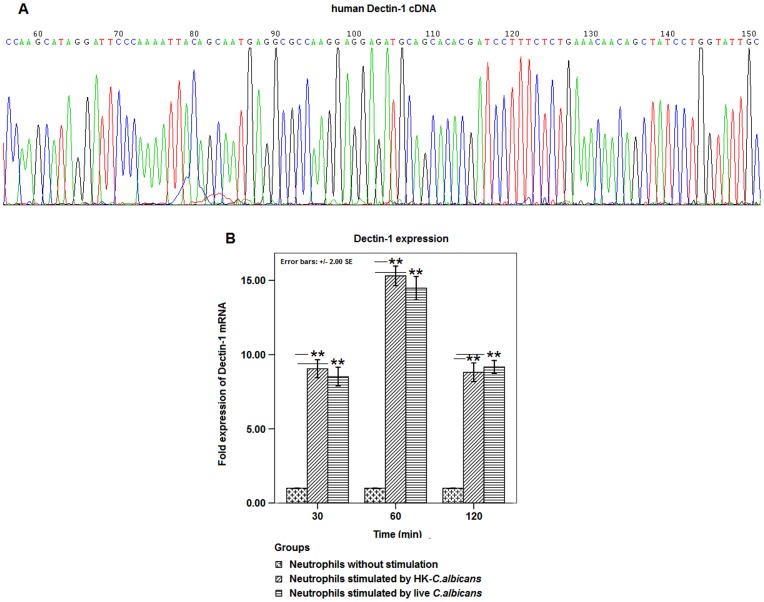
MBL*-*pre-incubated *C. albicans* stimulated mRNA expression of Dectin-1 in human neutrophils. **A.** Products obtained by using human Dectin-1 cDNA primers were sequenced and compared to the reference sequence in NCBI. **B.** Bar graph depicted the fold expression of neutrophil Dectin-1 mRNA at indicated time points after stimulation by live or HK-*C. albicans* at a MOI of 10 which was pre-incubated with 10 µg/mL MBL for 30 min at 37°C. Data were represented as mean ± SE (n = 20). ** Highly significant (P<0.01).

Sequence analysis of the amplified products obtained by using human Dectin-1 cDNA primers showed that the amplified region has 100% sequence identity to the reference sequence of Dectin-1 cDNA (NM_197954) in NCBI (Figure 3A). In comparison with the initial level, Dectin-1 expression at mRNA level was significantly up-regulated at 30 min (p<0.01) and reached the peak at 60 min (p<0.01) after stimulation by live or HK-*C. albicans* which was pre-incubated with MBL (Figure 3B). However, a decreased expression of Dectin-1 mRNA was observed at 120 min after stimulation (Figure 3B).

**Figure 4:**
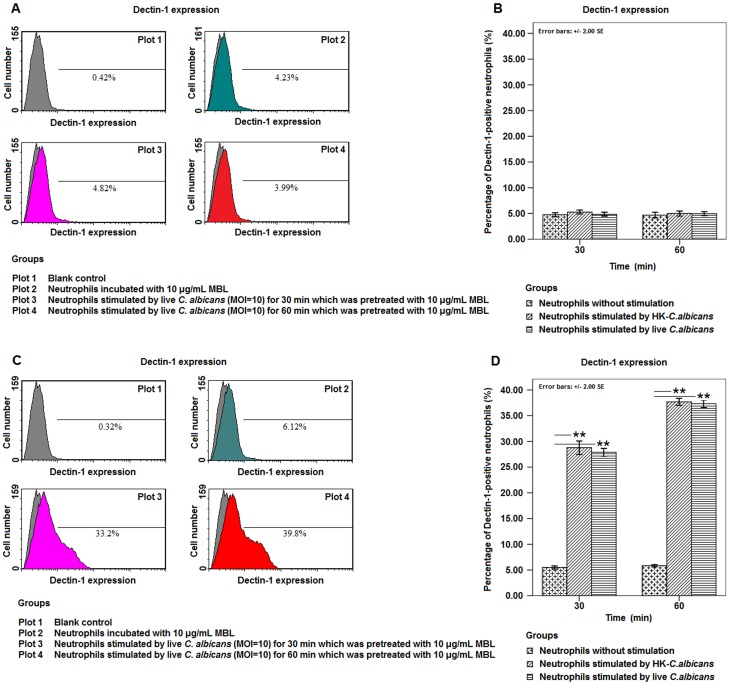
MBL*-*pre-incubated *C. albicans* stimulated intracellular expression of Dectin-1 in human neutrophils. The expression of neutrophil Dectin-1 was measured by PE-anti-human Dectin-1 mAb using flow cytometry at indicated time points after stimulation by live or HK-*C. albicans* at a MOI of 10 which was pre-treated with 10 µg/mL MBL. Mouse IgG2b was used as an isotype control. **A.** *C. albicans*-stimulated neutrophils were fixed with 1% paraformaldehyde, and the expression of Dectin-1 was measured by flow cytometry. **B.** Bar graph depicted the expression of Dectin-1 in neutrophils which were fixed with 1% paraformaldehyde at indicated time points after stimulation. **C.** After *C. albicans*-stimulated neutrophils were permeabilized and fixed with BD Cytofix/cytoperm solution for measuring intracellular cytokines, the expression of Dectin-1 was measured by flow cytometry. **D.** Bar graph depicted the expression of Dectin-1 in neutrophils which were permeabilized and fixed with BD Cytofix/cytoperm solution at indicated time points after stimulation. Data were represented as mean ± SE (n = 20). ** Highly significant (<0.01).

For human neutrophils fixed with 1% paraformaldehyde, flow cytometry assay for Dectin-1 showed that the percentage of Dectin-1-positive neutrophils remained almost unchanged at 30 or 60 min after stimulation by live or HK-*C. albicans* pre-incubated with MBL as compared to initial level (Figure 4A and 4B). For neutrophils permeabilized and fixed with Cytofix/cytoperm solution, which is specially used to measure intracellular cytokines, the assay showed that the percentage of Dectin-1-positive neutrophils increased significantly at 30 min and 60 min (p<0.001) after stimulation as compared to the initial level (Figure 4C and 4D).

### Blockage of Dectin-1 partly inhibited *C. albicans*-stimulated ROS production in neutrophils in the presence of exogenous MBL

**Figure 5:**
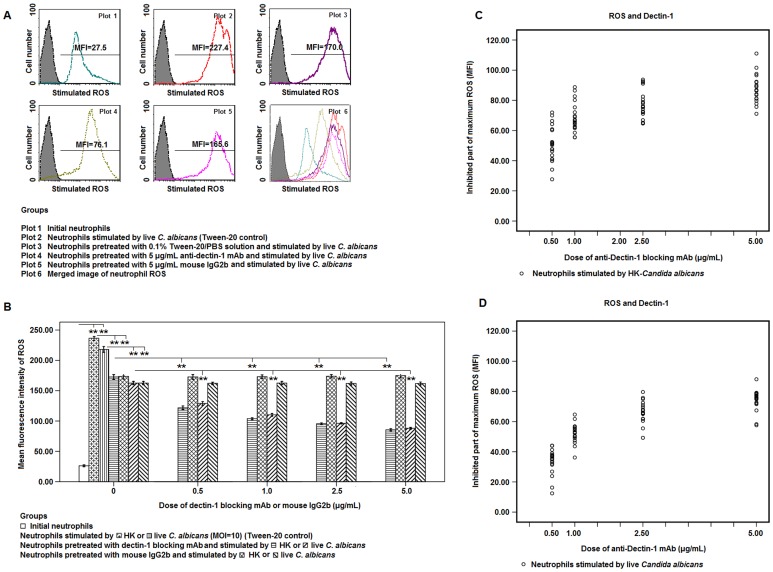
Abrogation of Dectin-1 partly inhibited ROS production in neutrophils which was stimulated by *C. albicans* in the presence of MBL. **A.** Following the pretreatment with 0.1% Tween-20/PBS solution containing 10 µg/mL of MBL and 5 µg/mL of anti-human Dectin-1 blocking mAb or the mouse IgG2b (Isotype control), the maximum value of intracellular ROS in neutrophils stimulated by live *C. albicans* (MOI = 10) was determined by flow cytometry during 120 min. The neutrophils treated with PBS containing 10 µg/mL of MBL were set as the Tween-20 control. The expression of ROS was represented as mean fluorescence intensity (MFI). **B.** Bar graph depicted the maximum value of intracellular ROS during 120 min after stimulation by live or HK-*C. albicans* in the presence of 10 µg/mL of MBL and different dosages of Dectin-1 blocking mAb or the isotype. Data were represented as mean ± SE (n = 20). ** Highly significant (<0.01). **C** and **D.** Linear regression analysis between HK- or live *C. albicans*-stimulated ROS in human neutrophils and the dosage of anti-human Dectin-1 blocking mAb * Significant (P<0.05), ** highly significant (<0.01).

Flow cytometry assay for ROS showed that the ROS generation by neutrophils reached the peak at 50 min to 80 min after phagocytosis and then decreased (data not shown). Statistical analysis showed that the maximum value of ROS stimulated by live or HK-*C. albicans* (MOI = 10) was significantly decreased in the abrogation groups in the presence of 10 µg/mL MBL when compared with that in untreated or mouse IgG2b-treated group, although it is still higher than the initial level (P<0.01, Figure 5A and 5B). In addition, the stimulated ROS in neutrophils pretreated only with PBS containing 0.1% Tween-20 was significantly lower than that in untreated neutrophils (Tween-20 control) (P<0.01, Figure 5A and 5B).

Linear regression analysis further suggested that the maximum of ROS generation in neutrophils was partly and irreversibly inhibited by Dectin-1 blocking mAb in a dose-dependent manner after stimulation by HK-*C. albicans* (R^2^ = 0.536, P<0.05, Figure 5C) or by live *C. albicans* (R^2^ = 0.668, P<0.05, Figure 5D).

### Intracellular Dectin-1 and ROS were recruited to the surface of phagocytized *C. albicans*

**Figure 6:**
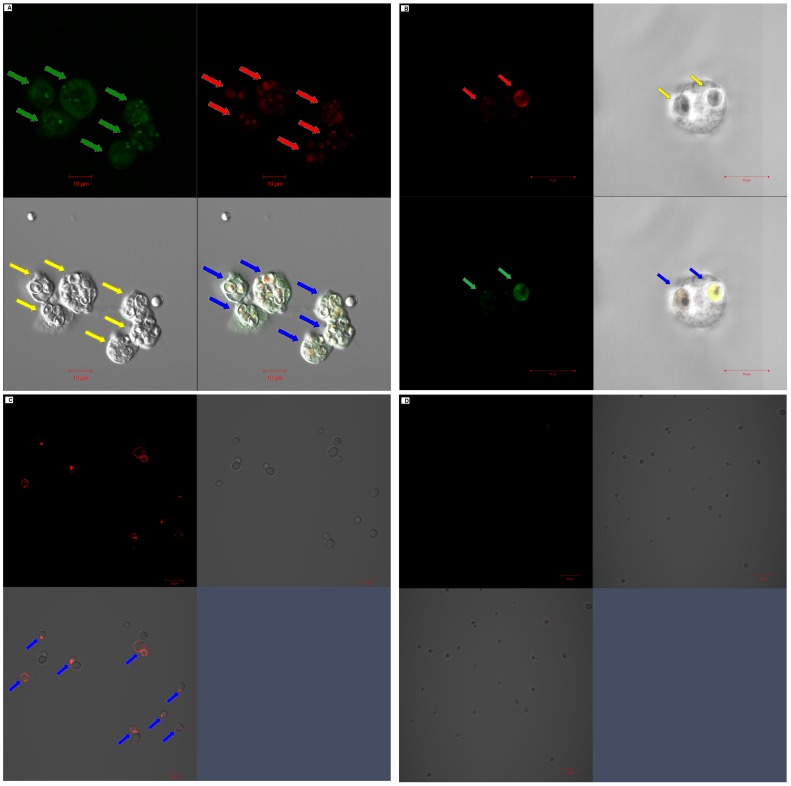
Intracellular expression of Dectin-1 was co-distributed with stimulated ROS on the *C. albicans* which was pre-incubated with MBL and phagocytized by human neutrophils. **A** **and B**. Localization of Dectin-1 expression and stimulated ROS were determined using confocal microscopy in human neutrophils which phagocytized live *C. albicans*. The distribution of intracellular Dectin-1 was indicated by red arrows and ROS by green arrows. The neutrophils which phagocytized *C. albicans* spores were indicated by yellow arrows. The localization association of intracellular Dectin-1 and stimulated ROS was indicated by blue arrows in the merged fluorescence image. **C.** rhDectin-1 binding before the treatment with *β*-1,3-glucanase. **D.** rhDectin-1 binding after the treatment with *β*-1,3-glucanase. **C** and **D**. Exposure of *β*-1,3-glucan on the spores of *C. albicans* was confirmed by indirect immunofluorescence assay using hrDectin-1 and PE-anti-human Dectin-1 mAb, and *β*-1,3-glucanase digestion test. The binding of Dectin-1 on the cell wall of *C. albicans* was indicted by blue arrows in the merged image. The data represented 3 similar experiments. Scale bars, **A, B** and **C.** 10 µm; **D.** 20 µm.

Confocal Laser Scanning Microscopy assay showed that the majority of intracellular Dectin-1 was recruited to the surface of some *C. albicans* spores which were phagocytized by neutrophils and partly co-colonized with the stimulated ROS (Figure 6A and 6B). Indirect immunofluorescence assay using rhDectin-1 and PE-anti-human Dectin-1 mAb showed that the binding site of rhDectin-1 was mainly distributed on the cell surface of *C. albicans* (Figure 6C). After *C. albicans* was pretreated with *ß*-1, 3-glucanase, which was used to specifically digest *ß*-1, 3-glucan moiety on the cell wall of *C. albicans*, the binding of human Dectin-1 on the surface of *C. albicans* nearly disappeared (Figure 6D).

## Discussion

MBL was well documented to have the ability to bind with high affinity to the mannose moiety of *Candida* species, and activate the complement system in complex with MASP, and greatly accelerate the C3/C4-mediated opsonophagocytosis of fungal pathogens [5], [6], [17], [18]. In this study, we demonstrated that the inhibitory effect on the phagocytosis of *C. albicans* by neutrophils caused by blockage of Dectin-1 was compensated by the exogenous MBL in a dose-dependent manner (Figure 2A–2F). This strongly suggested that MBL alone was also able to mediate the opsonophagocytosis of *C. albicans* by human neutrophils independent of complement activation which, to some extent, supported the role of neutrophil CR1 (CD35) as a cellular receptor for the collectin MBL. In agreement with the conclusion drawn by Deleo, *et al* that human neutrophils can effectively phagocytize the serum-opsonized *C. albicans* in a manner independent of Dectin-1 [4], our demonstration mentioned above further indicated that MBL may play a direct role in mediating the opsonophagocytosis of *C. albicans* by neutrophils via receptors other than Dectin-1.

In addition, this study evidenced that the transcriptional expression of Dectin-1 was up-regulated in the neutrophils after stimulation by *C. albicans* which was pre-incubated with exogenous MBL (Figure 3B). Interestingly, although Dectin-1 was traditionally considered to be a kind of type II transmembrane receptor which was expressed on the cellular membrane of innate immune cells [4], [9], [11], [12], our results indicated that the up-regulated expression of Dectin-1 was mainly distributed intracellularly, but not on the surface of neutrophils which was stimulated by MBL-pretreated *C. albicans* (Figure 4A–4D). More importantly, it was observed that the majority of intracellular Dectin-1 was recruited and co-distributed with the stimulated ROS on the surface of some *C. albicans* spores which were phagocytized by neutrophils (Figure 6A and 6B).

Although the treatment with 0.1% Tween-20/PBS solution, to some extent, decreased the maximum value of *C. albicans*-stimulated ROS in neutrophils, the abrogation test suggested that the stimulated ROS was further inhibited partly and irreversibly by blockage of Dectin-1 even if high-dose MBL was added (Figure 5A and 5B), while the inhibition of Dectin-1-mediated non-opsonic phagocytosis of *C. albicans* by neutrophils was completely compensated by the exogenous MBL (Figure 2D).

Furthermore, the results of digestion test using *β*-1, 3-glucanase also suggested that the specific binding of Dectin-1 with *β*-1, 3-glucan component on the cell wall of *C. albicans* could initiate the generation of intracellular ROS.

It is widely accepted that Dectin-1 plays a pivotal role in mediating the non-opsonic phagocytosis of fungal pathogens by human neutrophils including *Aspergillus fumigatus* conidia, *Saccharomyces cerevisiae*, *Candida albicans* [4], [9], [11], [12], [19]. Recent investigations have further elucidated that Dectin-1-mediated phagocytosis can activate the Syk-mediated signaling pathway by its ITAM-containing intracellular tail, which takes part in the intracellular ROS initiation [12], [20], [21]. Noticeably, the findings in this study suggested that Dectin-1-triggered ROS generation should be considered as an independent and indispensable biological behavior in the fungicidal process of neutrophils, which could collaborate with the MBL-dependent opsonophagocytosis via receptors other than Dectin-1. Moreover, this possible coupling will, to some extent, facilitate the killing of *C. albicans* in the bloodstream, and therefore improve host defense against hematogenously disseminated candidiasis.

On the other hand, our finding of partial inhibition of ROS production by the abrogation of Dectin-1 suggested that the receptors other than Dectin-1 may play a role in the ROS production, and the signaling cross-talk between different pattern recognition receptors will determine the ROS-dependent fungicidal activity of human neutrophils.

In summary, the present study extends our understanding of biological behavior of Dectin-1 as well as the ROS-dependent fungicidal activity of neutrophils partly mediated by Dectin-1. It is rational to explore further in future research on whether CR1 functions as a cellular receptor of MBL during the process of MBL-mediated phagocytosis of *C. albicans* by neutrophils, and couples with intracellular Dectin-1-triggered ROS production.

## Materials and Methods

### Chemical Reagents and antibodies

Histopaque 1077 (Product No: 10771, Sigma-Aldrich) and Histopaque 1119 (Product No: 11191, Sigma-Aldrich) were used for the isolation of human neutrophils. Recombinant human MBL (Cat No: 2307-MB, R&D systems) was used for the pre-incubation. Fluorescein isothiocyanate isomer I (FITC) (Cat No: F7250, Sigma-Aldrich) was used to label *C. albicans* for phagocytosis. For Dectin-1 mRNA assay, a Trizol Reagent Kit (Cat No: 15596-026, Invitrogen Life technologies), a First Strand cDNA Synthesis Kit (ReverTra Ace-α-, Cat NO: FSK-100, TOYOBO) and a THUNDERBIRD SYBR qPCR Mix (Cat NO: QPS-201, TOYOBO) were used.

For Dectin-1 assay by flow cytometry, BD Cytofix/Cytoperm solution (Cat No: 554723, BD Biosciences) was used to fix and permeabilize human neutrophils before staining for intracellular Dectin-1. Phycoerythrin (PE) conjugated anti-human Dectin-1 monoclonal antibody (Clone#: 259931, Catalog No: FAB1859P, R&D systems) was used to detect Dectin-1 expression on human neutrophils. PE conjugated mouse IgG2b (Clone#: 133303, Catalog No: IC0041P, R&D systems) was used as an isotype control antibody.

For antibody inhibition experiment, mouse anti-human Dectin-1 blocking antibody (Clone# 259931, Catalog No: MAB1859, R&D systems) was used to block human Dectin-1 receptor, and mouse IgG2b (Clone#: 20116, Catalog No: MAB004, R&D systems) was used as an isotype control antibody.

In addition, 2′,7′-Dichlorofluorescein diacetate (DCFH-DA, CAS-No: 2044-85-1, Sigma) was used for ROS assay. Recombinant Human Dectin-1/CLEC7A (Catalog No: 1859-DC) and *β*-1, 3-D-glucanase from *Helix pomatia* (CAS No: 9044-93-3, Sigma, China) were used for *β*-1, 3-glucan digestion test.

### Isolation of human neutrophils

The study was approved by the Institutional Review Board, and was considered in compliance with the Ethics Requirements. Human neutrophils were obtained from fresh venous blood of 20 healthy individuals according to a protocol approved by the Institutional Review Board for Human Subjects, National Institute of Health. Peripheral blood was collected in Sodium Citrate tubes (9∶1) and the neutrophils were separated by a standard technique of density-gradient centrifugation over Ficoll-Hypaque as described previously [17]. The purity of human neutrophils isolated from peripheral blood was detected by Wright Stain, which can distinguish easily between blood cells, and was used primarily to stain peripheral blood smears.

### Preparation of heat-killed and FITC-labeled *C. albicans*

*C. albicans* (ATCC 10231) was maintained on Sabouraud dextrose agar (Difco Laboratories, Detroit, Mich.) by passages and was grown for 16 hours in Sabouraud dextrose broth at 30°C with orbital shaking at 150 rpm. Heat-killed (HK-) *C. albicans* was prepared by adjusting the cell density to 2×10^7^/mL and kept at 65°C in a water bath for 1 hour [18].

FITC-labeled *C. albicans* was prepared by labeling the fresh HK-*C. albicans* with FITC according to the methods described previously [6], [18]. The homogeneous distribution of FITC-*C. albicans* was confirmed by flow cytometry assay.

### Antibody inhibitory experiments

To block the Dectin-1 receptor, human neutrophils (2×10^6^/mL) were incubated in PBS containing 0.1% Tween-20 and different concentrations of the blocking antibody at 0.5, 1, 2.5 and 5 µg/mL, respectively, for 60 min at 4°C. The cells were then washed and resuspended in PBS. The human neutrophils treated with mouse IgG2b and the initial neutrophils were used as controls. The performance of abrogation was detected by flow cytometry assay.

### Measurement of phagocytic activity of human neutrophils after Dectin-1 blockage in the presence of exogenous MBL

The exogenous MBL at different concentrations (2.5, 5.0 and 10.0 µg/mL) was added respectively into the neutrophils (2×10^6^/mL) that had been pretreated with 5 µg/mL Dectin-1 blocking mAb for 60 min at 4°C. The neutrophils untreated and these only treated with Dectin-1 blocking mAb were set as controls. Afterwards, the neutrophils in different groups were incubated with an equal volume of FITC-*C. albcians* (MOI = 10) for 30 or 60 min at 37°C, and the phagocytosis efficiencies were determined by flow cytometry as described previously.

### Measurement of Dectin-1 expression in neutrophils after stimulation by *C. albicans* pre-incubated with MBL

The neutrophils were subjected to stimulation for 30, 60 or 120 min by live or heat-killed *C. albicans* (MOI = 10) which was pre-incubated with 10 µg/mL MBL for 30 min at 37°C. Expressions of neutrophil Dectin-1 at mRNA and cellular levels were measured by Real-time quantitative PCR and flow cytometry assay respectively.

For the measurement of Dectin-1 mRNA in neutrophils, Trizol Reagent Kit was used to extract the whole RNA component from human neutrophils. TOYOBO First Strand cDNA Synthesis Kit and THUNDERBIRD SYBR qPCR Mix were introduced to synthesize the first-strand cDNA and initialize the Real-Time quantitative RT-PCR. The procedures were done according to the kit protocols. Primers specific to Dectin-1 cDNA and *β*-actin (internal control) were designed as follows:

Human-Dectin-1-S: 5′-GCAATACCAGGATAGCTGTTG-3′;

Human-Dectin-1-A: 5′-CCAAGCATAGGATTCCCAA-3′.

Human-*β*-actin-S: 5′- GTCCACCGCAAATGCTTCTA-3′;

Human-*β*-actin-A: 5′- TGCTGTCACCTTCACCGTTC-3′.

SLAN Real-Time quantification PCR detection system was applied in this study and the results were analyzed by relative quantification CT values & 2-ΔΔCT method.

For the cell surface Dectin-1 assay, the neutrophils after stimulation were washed three times with PBS (pH = 7.2), and then fixed with 250 µL of 1% paraformaldehyde at 4°C for 20 min. For the intracellular Dectin-1 assay, BD Cytofix/Cytoperm solution was used to fix and permeabilize the cells according to the protocol provided by BD Bioscience. The cell pellets were then washed for three times with PBS solution and incubated with 10 µL PE-anti-human Dectin-1 monoclonal antibody or isotype control antibody for 45 min at 4°C.

Following the incubation, the cells were washed three times with PBS (pH = 7.2) prior to flow cytometry analysis. The neutrophil can be clearly differentiated from *C. albicans* by flow cytometry based on their different characteristics reflected by forward scatter (FSC) and side scatter (SSC). The group in which the neutrophils were treated with PE-labeled mouse IgG2b was set as isotype control.

### Measurement of stimulated ROS in neutrophils after Dectin-1 blockage in the presence of MBL

0.5 mL neutrophils (2×10^6^/mL) were pretreated with 0.1% Tween-20/PBS solution containing 10 µg/mL MBL and different concentrations of blocking antibody or the isotypes (mouse IgG2b) for 60 min at 4°C as mentioned in “Antibody inhibitory experiments” section, and then with 25 µM 2′,7′-Dichlorofluorescein diacetate (DCFH-DA) for another 20 min at room temperature. The group pretreated only with PBS containing 10 µg/mL MBL was set as Tween-20 control. Afterwards, the neutrophils were respectively stimulated by 0.5 mL of live or HK-*C. albicans* (MOI = 10). The rate of ROS production over time was measured at 10-min intervals for up to 120 min by flow cytometry.

### Flow cytometry assay

In the present study, the flow cytometer (Epics Altra II, Beckman Coulter, USA) was used to detect the expression of neutrophil Dectin-1, the phagocytosis of FITC-*C. albicans* by human neutrophils, and the intracellular ROS production. The excitation and emission wavelengths of 488 and 525 nm were used for FITC or ROS assay, and 488 and 585 nm for PE assay, respectively. The data were analyzed using Expo32 v1.2.

### Co-distribution examination of intracellular ROS and Dectin-1 in the presence of MBL

The neutrophils (2×10^6^/mL) were incubated with PBS containing 25 µM of DCFH-DA for 20 min at room temperature, and stimulated at 37°C for another 60 min by live or HK-*C. albicans* (MOI = 10) which were pretreated by 10 µg/mL of MBL as mentioned above. The cells were treated with 0.1% Tween-20 for 20–30 min at 4°C and then washed twice with PBS. Subsequently, 10 µL of PE-anti-human Dectin-1 was added and the cell pellets (50 µL) were incubated at 4°C for 40 min. The localizations of Dectin-1 and ROS were observed by Confocal/two-photon Laser Scanning Microscopy (LSM710, Zeiss). The excitation wavelength was set as 488 nm, and the emission wavelength was set as 525 nm for the ROS assay or 585 nm for the PE assay, respectively. The cells treated with the PE-mouse IgG2b or with DCFH-DA alone were used to set fluorescence compensation.

### *β*-1, 3-glucanase digestion test

The indirect immunofluorescence assay was used to identify the exposure of *β*-1, 3-glucan on the cell wall of live *C. albicans*. Briefly, 0.5 mL of *C. albicans* suspension (1.0×10^7^ CFU/mL) was blocked with 3% bovine serum albumin (BSA) at 20°C for 30 min and then incubated with the human derived Dectin-1 (10 µg/mL, Cat No: 1859-DC) at 4°C for 1 hour. The cells were then washed with PBS (pH = 7.2) for three times and incubated with the PE-anti-human Dectin-1 at 4°C for 45 min. In addition, *β*-1,3-D-glucanase from *Helix pomatia* (CAS No: 9044-93-3, Sigma, China mainland) was used to identify whether the specific recognition and binding site of human Dectin-1 was the *β*-1, 3-glucan moiety on the cell wall of live *Candida albicans*. Briefly, the suspension of *C. albicans* was pre-incubated with *β*-1,3-D-glucanase (10 U/mL) at 37°C for an hour and then washed with PBS (pH = 7.2) for three times prior to the indirect immunofluorescence assay. The Confocal Laser Scanning Microscopy was used to analyze the specific recognition and binding of hrDectin-1 with the *β*-1, 3-glucan moiety on the cell wall of *C. albicans*.

### Statistics

Univariate ANOVA for compatibility groups design, LSD-t test and linear regression analysis were applied for statistical analysis by using SPSS. 13.0 software. P values of 0.05 or less were considered significant.
